# Wetlands, wild Bovidae species richness and sheep density delineate risk of Rift Valley fever outbreaks in the African continent and Arabian Peninsula

**DOI:** 10.1371/journal.pntd.0005756

**Published:** 2017-07-25

**Authors:** Michael G. Walsh, Allard Willem de Smalen, Siobhan M. Mor

**Affiliations:** 1 Marie Bashir Institute for Infectious Diseases and Biosecurity, University of Sydney, Westmead, New South Wales, Australia; 2 Westmead Institute for Medical Research, University of Sydney, Westmead, New South Wales, Australia; 3 School of Public Health, University of Sydney, Camperdown, New South Wales, Australia; 4 School of Veterinary Science, Faculty of Science, University of Sydney, Camperdown, New South Wales, Australia; Institute for Disease Modeling, UNITED STATES

## Abstract

Rift Valley fever (RVF) is an emerging, vector-borne viral zoonosis that has significantly impacted public health, livestock health and production, and food security over the last three decades across large regions of the African continent and the Arabian Peninsula. The potential for expansion of RVF outbreaks within and beyond the range of previous occurrence is unknown. Despite many large national and international epidemics, the landscape epidemiology of RVF remains obscure, particularly with respect to the ecological roles of wildlife reservoirs and surface water features. The current investigation modeled RVF risk throughout Africa and the Arabian Peninsula as a function of a suite of biotic and abiotic landscape features using machine learning methods. Intermittent wetland, wild Bovidae species richness and sheep density were associated with increased landscape suitability to RVF outbreaks. These results suggest the role of wildlife hosts and distinct hydrogeographic landscapes in RVF virus circulation and subsequent outbreaks may be underestimated. These results await validation by studies employing a deeper, field-based interrogation of potential wildlife hosts within high risk taxa.

## Author summary

Rift Valley fever (RVF) is a vector-borne zoonotic disease that imparts a substantial burden to the economy and public health of pastoralist communities across the African continent and Arabian Peninsula. Furthermore, RVF is also an emerging pathogen of growing global concern. Knowledge of the epidemiological and ecological factors that influence the geographic distribution of RVF outbreaks and determine risk for humans and animals is incomplete. The current study examined the distribution of RVF outbreaks from 1998 to 2016 and modeled their occurrence as a function of climate, surface water, land cover, livestock density, wild mammalian species richness, and human migration. The results indicate that wetlands, Bovidae species richness, and sheep density were associated with increased risk of RVF outbreaks. Our findings contribute to improved understanding of the spatial and ecological dynamics of RVF risk with a particular emphasis on the distribution of wetlands and potential wildlife reservoirs in designing RVF surveillance programs.

## Introduction

Rift Valley fever (RVF) is an emerging, vector-borne viral zoonosis that causes significant morbidity in humans and their livestock. The etiologic agent, Rift Valley fever virus (RVFV), is a Phlebovirus in the *Bunyaviridae* family and is transmitted by several mosquito species that facilitate viral maintenance (*Aedes* spp.) or amplification (*Culex* spp.) [1,2]. Human infections are invariably asymptomatic or mild in early stages, however, severe cases can manifest as hemorrhagic fever or encephalitis [3,4]. Sheep, goats, and cattle experience fetal abortions as a result of RVFV infection, and the disease contributes to substantive economic losses to pastoralist communities during outbreaks [5,6].

Historically, most outbreaks in humans and domestic animals have occurred in the African continent, and in eastern Africa these typically follow periods of excessive rain in poorly draining arid or semi-arid landscapes [7]. The resultant flooding is conducive to the breeding and hatching of infected mosquitoes which transmit the virus to ruminant hosts followed by eventual secondary transmission to other ruminants and humans [5]. In more recent years, RVF has progressively expanded east into the Arabian Peninsula, with outbreaks in Saudi Arabia and Yemen [8].

The epidemiology and infection ecology of RVFV is complex and our knowledge of these incomplete. Several species in two distinct mosquito genera transmit infection. As primary vectors, *Aedes* mosquitoes maintain RVFV transovarially during dry periods; during these times there are little to no reported human or livestock infections. *Aedes* mosquito population explosion following wet periods leads to localized transmission to mammalian hosts [9,10]. Following this, *Culex* mosquitoes can expand (amplify) transmission to more dispersed livestock and human populations distant from the areas of local *Aedes* transmission [2,11,12]. Once RVFV becomes amplified in livestock, ongoing human infection occurs primarily through zoonotic transmission as a result of direct or indirect contact with animal tissues and body fluids, such as occurs during slaughtering or through performing obstetrical procedures on infected animals. Transmission from mosquitoes that feed on infected animals is also a viable though less important source of human infection [1,13].

While the role of vectors in RVFV infection ecology is well-established, the extent to which wildlife contributes to transmission as possible maintenance or amplification hosts is not well understood. Field investigations suggest that wild ruminants and rodents are the most likely RVFV reservoirs [14]. Nevertheless, data from these field surveys are limited, so definitive mammalian natural reservoirs for RVFV are not described [11,14].

The landscape epidemiology of RVFV is also incomplete with respect to abiotic systems of influence. For example, periods of excessive rain are strongly associated with RVF outbreaks in East Africa [7,15–18], however very little is known regarding the interaction between climate and terrestrial or hydrogeographic profiles in mediating RVF outbreaks [19]. In addition, there has been a lack of attention to land cover characteristics, which have the potential to influence mosquito habitat, sylvan reservoir habitat, and the movement of domestic livestock through the landscape. Finally, anthropogenic influence, such as human migration, may introduce novel, or increase existing, exposures among pastoralist and/or other rural and peri-urban communities [20].

The study sought to expand our current understanding of RVF epidemiology and infection ecology by investigating the role of diverse hydrogeographic features and wild Bovidae and Muridae species richness in delineating the landscape suitability of future outbreaks across the African continent and Arabian Peninsula.

## Methods

### Data sources

Occurrence data for RVF outbreaks in humans and livestock animals were obtained from the ProMED-mail electronic surveillance system. This surveillance system is maintained by the International Society of Infectious Diseases and provides near real time and archival documentation of formal and informal reports of infectious diseases [21]. The database was searched using the keywords “rift valley fever”, “rift valley fever virus”, “rvf”, and “rvfv”. Only those reports documenting RVF outbreaks in humans or livestock in unique locations were included (i.e. duplicate outbreaks were not included). One hundred and three reports of laboratory confirmed, geolocated outbreaks of RVF in humans and livestock were documented by the ProMED system between January 1, 1998 and August 31, 2016. Google Maps was used to capture the geographic coordinates for each outbreak and cross-checked against Open Street Map. Centroids of the reported outbreak locations were recorded to a spatial resolution of 4km^2^.

To test our landscape suitability model (see Statistical Analysis section), a second source of RVF outbreak data were obtained from the World Organization for Animal Health (OIE). OIE maintains an official biosurveillance mechanism for RVF in livestock. These data have been archived since 2004 and can be accessed via the World Animal Health Information System (WAHIS) web portal [22]. Reports included the location of each event by place name, the date, type of livestock affected, and the number of infected animals identified. Between January, 2005 and August, 2016 a total of 50 "immediate notification" and subsequent “follow-up” RVFV outbreak reports were submitted to OIE. The geographic coordinates for these events were obtained with Google Maps as above. Outbreaks from ten of these reports could not be located within this coordinate reference system. This left a total 40 OIE reports with 102 unique outbreak occurrences. Twenty-three of the OIE documented outbreaks were also recorded in the ProMED surveillance and therefore were not included in this testing dataset to prevent inflation of model performance. Thus, the final OIE sample of 79 was used for model testing.

Altitude and four Bioclim climate rasters were obtained from the WorldClim Global Climate database and used as climate indicators for this investigation [23]. Aggregate spatio-temporal weather station data between 1950 and 2000 were used to calculate the mean temperature during the hottest and coldest quarters, and the mean precipitation during the wettest and driest quarters, and extracted as 30 arc second (approximately 1 km^2^) resolution rasters [24].

Vegetation cover was assessed using the MODIS-based Maximum Green Vegetation Fraction (MGVF), which is a data product from the United States Geologic Survey's Land Cover Institute [25]. The MGVF records the percentage of green vegetation cover per pixel as a function of the normalized difference vegetation index at a resolution of 1 km^2^[26]. Rasters were obtained at two time points, years 2001 and 2010, and the difference between them calculated to determine vegetation loss over this 10 year period. Change in MGVF over this time period was considered a more robust representation of vegetation cover than mean MGVF, and therefore more appropriate in assessing its influence on RVF landscape suitability.

The Global Lakes and Wetlands Database [27] was used to define surface water. This raster was derived from three discrete components. The first two comprised vector data of polygons. Component 1 represented lakes with area ≥ 50 km^2^ and controlled water reservoirs with volume ≥ 0.5 km^3^, while component 2 represented all surface water with area ≥ 0.1 km^2^. The third component combined and rasterized the polygon data from the first two components, while supplementing the wetland data. The final 1 km^2^ raster based on component three was used here. The surface water categories were: lake, controlled water reservoir, river, freshwater marsh, swamp, coastal wetland, brackish, bog, or intermittent wetland [28]. The surface water types were extracted and new distance rasters created. Distance was calculated in the QGIS geographic information system using the proximity function to produce separate 1 km^2^ resolution rasters for each water category[29]. Pixel values in these rasters convey the distance in kilometers between a given pixel and the nearest pixel occupied by each unique category of surface water. In this way the models can incorporate a spectrum of proximity to diverse hydrogeography across the metacontinent (see Statistical Analysis section).

Net human population migration was obtained as a 30 arc-second raster from the Socioeconomic Data and Applications Center (SEDAC), which is part of the National Aeronautics and Space Agency's Earth Observing System Data and Information System [30]. This raster describes the net change (increase vs. decrease) in persons per km^2^ from the period 1990 to 2000 [31].

The global densities of cattle, sheep, and goats were represented as 1 km^2^ resolution rasters from the Gridded Livestock of the World (GLW) [32]. The GLW also classified ruminant livestock production systems by system (livestock-only, mixed rain fed, and mixed irrigated) and climate regime (Hyper-arid, Arid, Humid, and Temperate/Tropical Highlands) comprising 12 production system categories plus one additional category classified as Urban [33].

Rasters of Bovidae and Muridae species richness at 1 km^2^ resolution were acquired from the International Union for Conservation of Nature (IUCN) and Center for International Earth Science Information Network (CIESIN)[34].

Finally, all species of *Aedes* mosquitoes observed across the geographic range of RVF outbreaks were extracted from the Global Biodiversity Information Facility (GBIF)[35]. There were 215 field observations of *Aedes* mosquitoes geolocated within the African continent and Arabian Peninsula. However, of these 215 mosquito observations, 151 observations recorded the genus only without species designation, while 57 were *Ae*. *africanus*, and 7 were *Ae*. *albopictus*. As such, there was not sufficient species representation in the GBIF to produce valid models of the ecological niche of *Aedes* vectors. One generic ecological niche model of *Aedes* mosquitoes was included in an exploratory analysis, but this contributed very little to the loss function when modeling RVF landscape suitability (see Statistical Analysis section), further suggesting that the mosquito data were insufficient to include in the current investigation. Similarly, this analysis did not include potential *Culex* amplification vectors as there were too few GBIF specimens across the region and those that were present were of too diverse an ecological and behavioral spectrum to be pooled for analysis.

### Statistical analysis

This study used maximum entropy (Maxent) machine learning to model the landscape suitability of RVF outbreaks in human and livestock hosts across Africa and the Arabian Peninsula at a resolution of 4 km^2^. In the current study, *risk* is defined explicitly as the probability of landscape suitability to RVF outbreaks. Machine learning in general, and Maxent in particular, is analytically appealing because a specific model form is not assumed. Instead algorithms create rule-based data partitions that optimize homogeneity between predictors and outcomes [36]. Further, the Maxent machine learning algorithm does not require the locations of RVF outbreak absences which are effectively unknowable [37,38].

The full Maxent model (based on ProMED data) comprised the following landscape features: mean dry quarter precipitation; mean warm and cold quarter temperature; change in vegetation cover; proximity to the surface water features; wild Bovidae and Muridae species richness; cattle, sheep, and goat densities; and net human migration between 1990 and 2000. Correlation between most of the landscape factors acquired for this study was low. However, there were a few exceptions (wet quarter precipitation, ruminant production systems, swamp, and altitude), all of which were correlated with several other landscape factors and provided generally redundant information. Therefore, these factors were dropped from the original 22 predictors acquired from our data sources described above. Ten thousand background points were sampled, weighted according to human population density to adjust for any potential sampling bias in RVF occurrences derived from ProMED. A value of 1.0 was selected for the regularization parameter, to correct for overfitting of the model predictions. The Maxent models were trained using five-fold cross-validation. This approach divides the training set into k = 5 subsets, iteratively fits the model to 4-subset combinations, and then tests against the 5^th^. Each of the five subsets included approximately 20 RVF outbreaks selected randomly from the total number of available observations in the training dataset (ProMed; n = 103 outbreaks).

Landscape features used in the full Maxent model were ranked according to their permutation importance, which randomly permutes the values of the landscape factors between background and presence points in the training dataset. This is preferred over the direct percent contribution to the loss function because it is non-heuristic and more robust to any residual correlation in assessing the influence of individual features on RVF landscape suitability [37,39].

Finally, as a robust evaluation of prediction error, the trained models were tested against the data obtained from OIE. The difference in model predictions based on the training and testing data was used to assess the model prediction error, which was reported as the area under the curve (AUC). The models were fit using the maxent function (dismo package; v. 0.9–3) setting the distribution to Bernoulli [38,40,41]. All analyses were performed using R statistical software version 3.1.3 [42].

## Results

**Fig 1:**
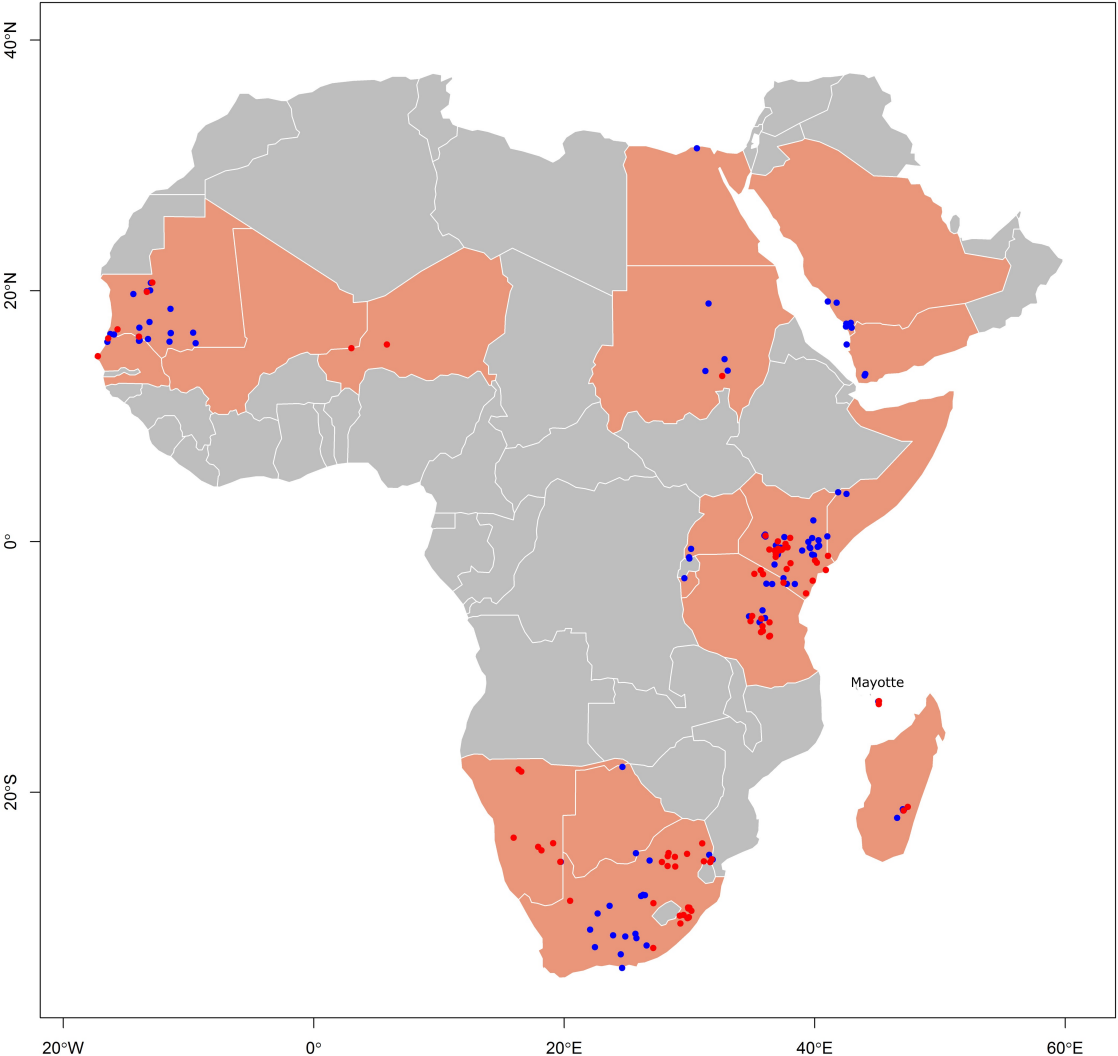
The distribution of Rift Valley fever outbreaks across the African continent and the Arabian Peninsula between 1998 and 2016 as reported by ProMED-mail (blue) and between 2005 and 2016 as reported by the World Organization for Animal Health (red). Countries affected are highlighted in salmon and include: Botswana, Burundi, Egypt, Kenya, Madagascar, Mali, Mauritania, Mayotte (France), Namibia, Niger, Saudi Arabia, Senegal, Somalia, South Africa, Sudan, Tanzania, Uganda, Yemen.

The distributions of RVF outbreaks captured by ProMED and OIE are presented in Fig 1. The clustering of these outbreaks in the Sahel and in eastern and southern African is demonstrated, as is the more recent emergence of RVFV in the Arabian Peninsula.

**Fig 2:**
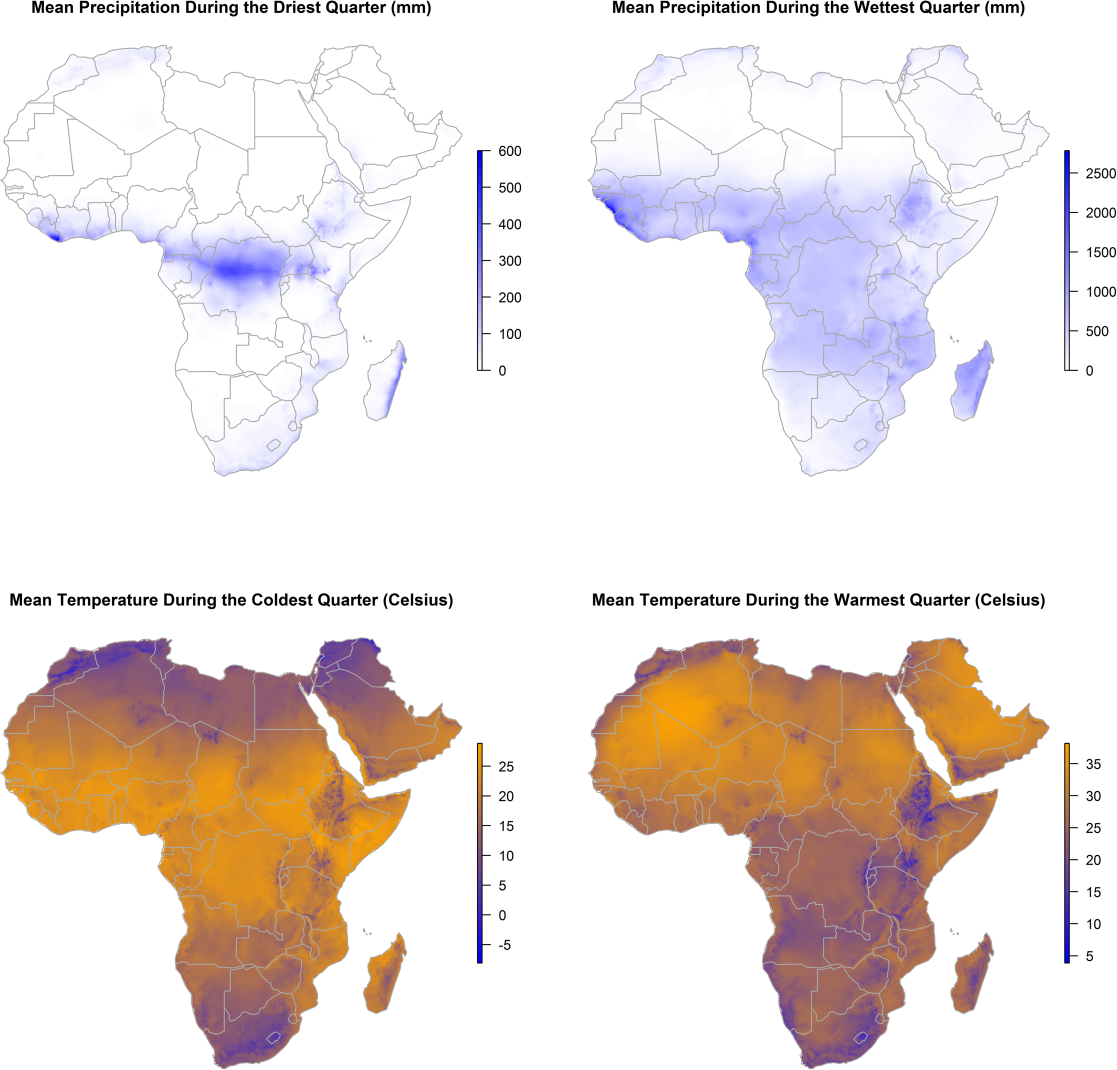
Distributions of driest and wettest quarter precipitation (upper panels) and coldest and warmest quarter temperature (lower panels) across Africa and the Arabian Peninsula.

**Fig 3:**
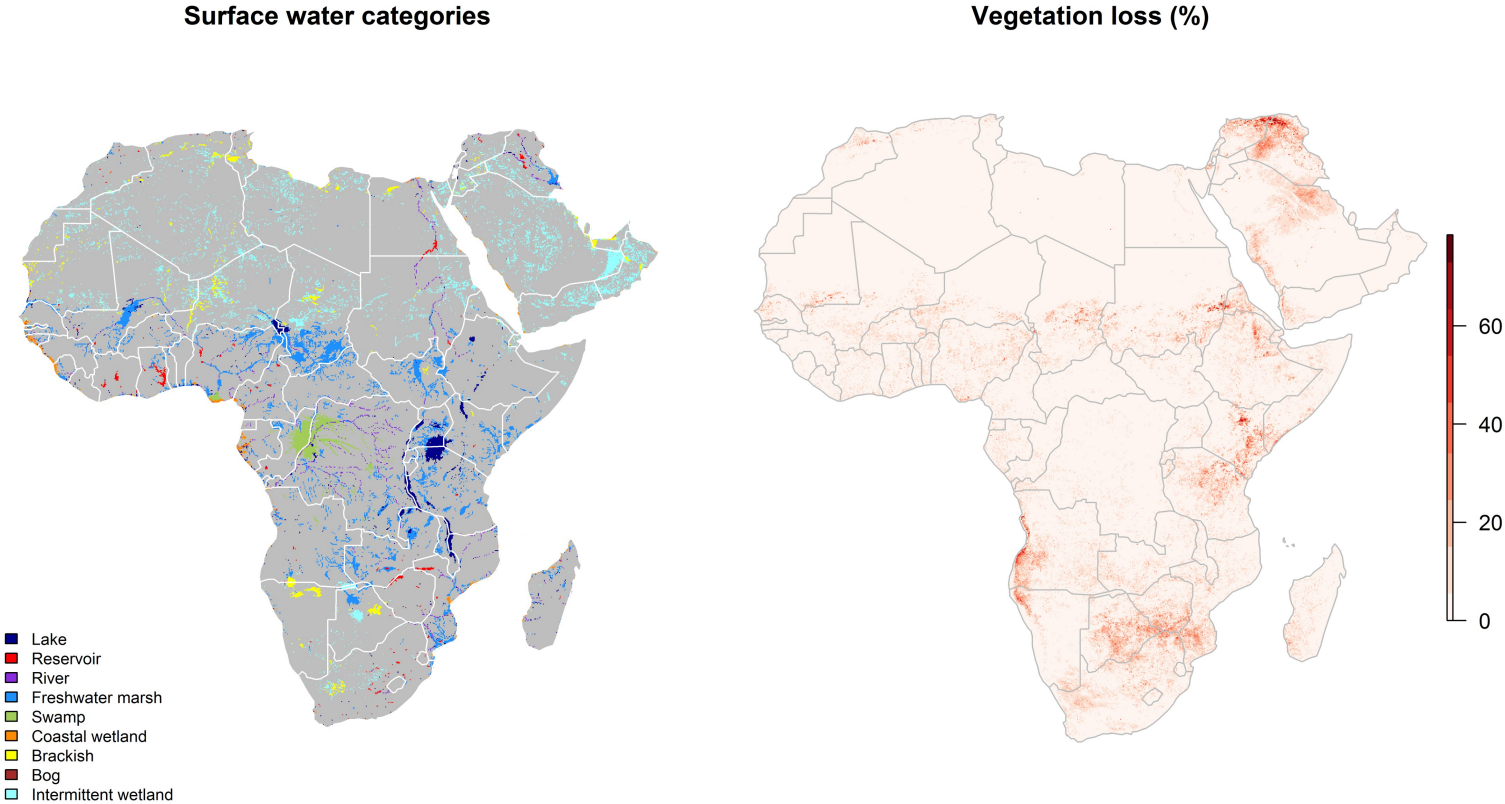
Distributions of surface water (left panel) and vegetation loss (right panel) across Africa and the Arabian Peninsula.

**Fig 4:**
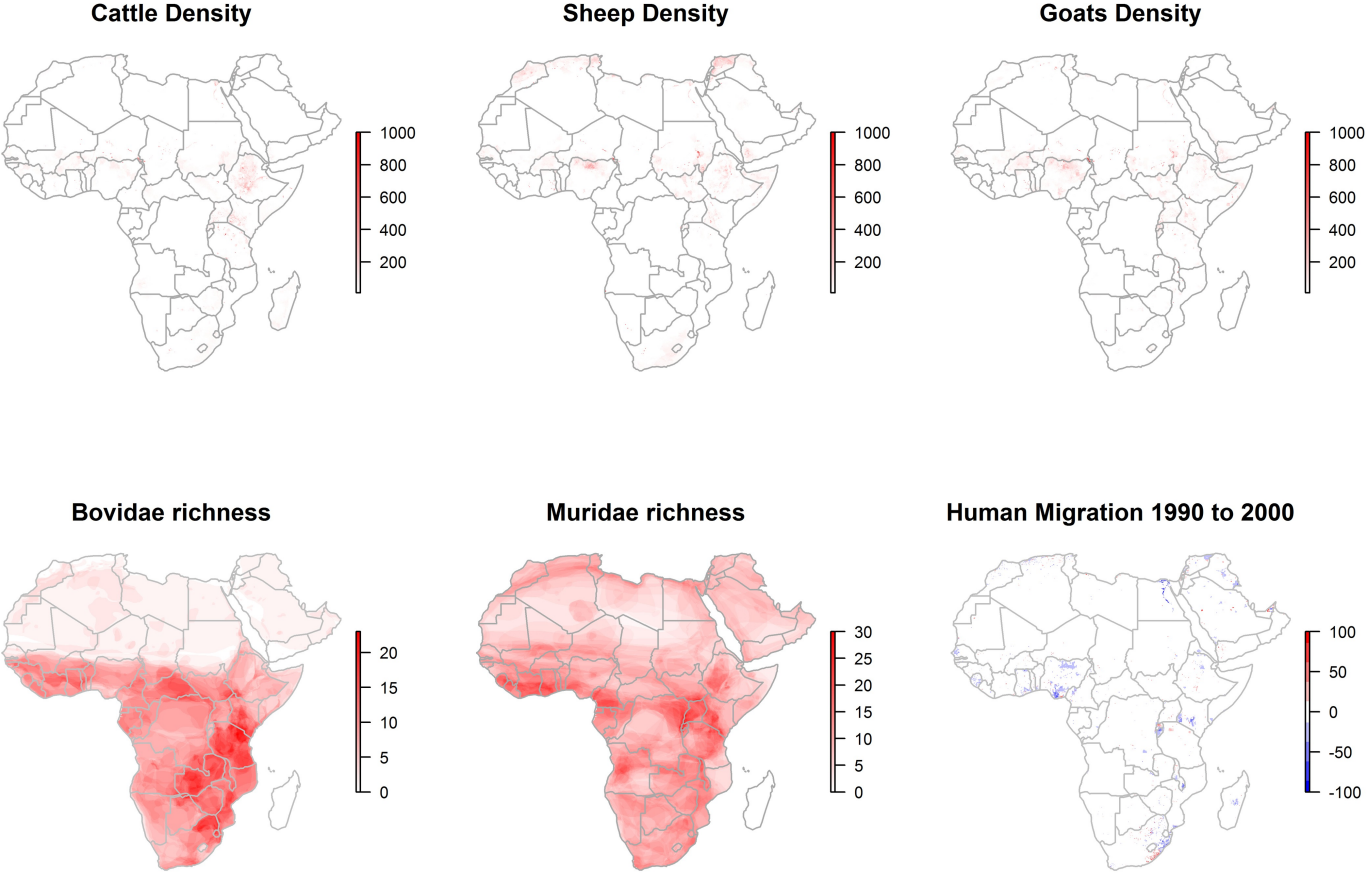
Distributions of livestock (animals per km^2^), wild Bovidae and Muridae species richness (number of species per km^2^), and net human migration (number of people into [red] or out of [blue] each km^2^).

All landscape features used in the ecological niche modeling are presented separately for the abiotic (climate, vegetation change, and surface water) and biotic (livestock densities, Bovidae and Muridae species richness, and human population migration) features in Figs 2, 3 and 4.

**Fig 5:**
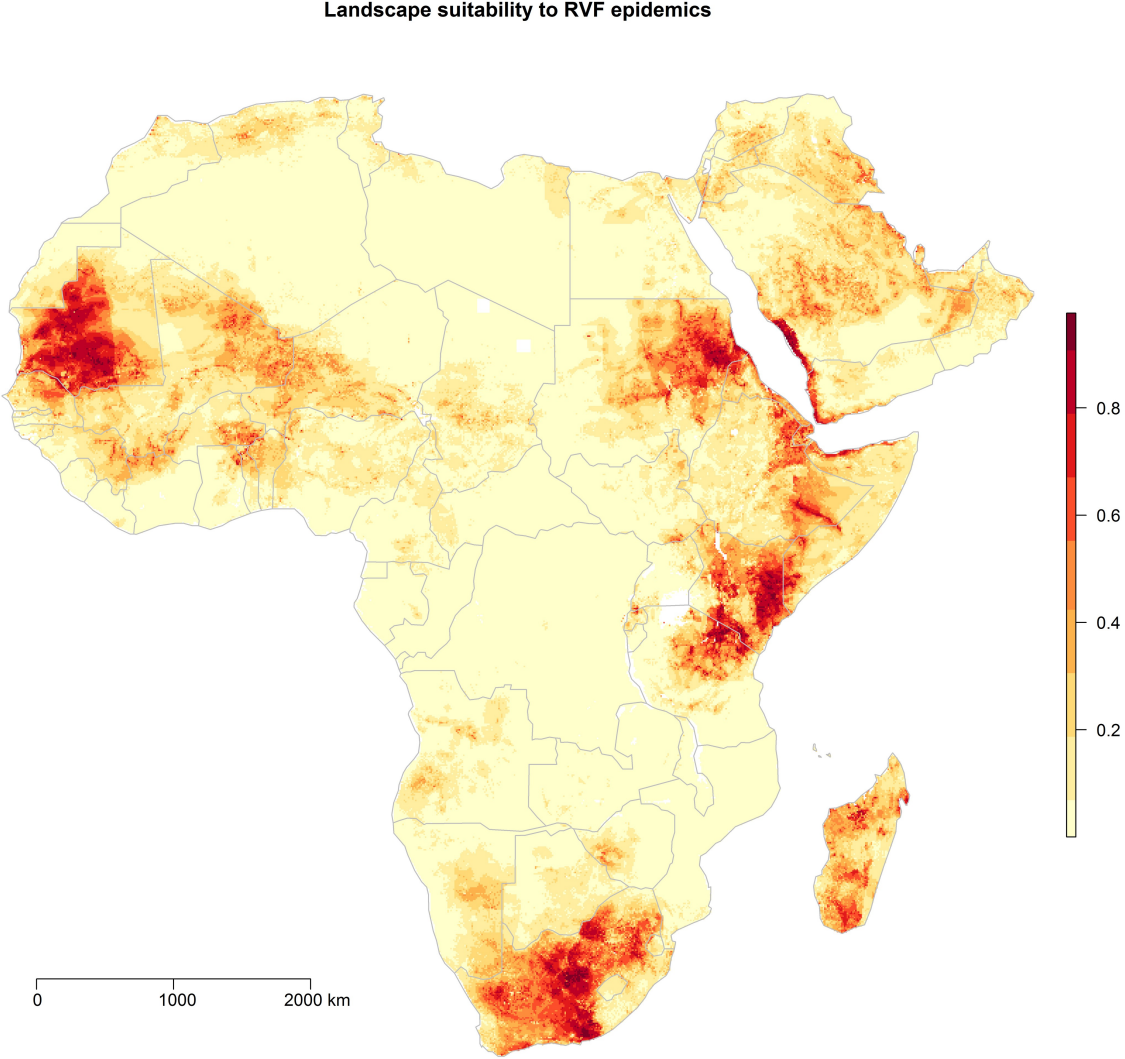
Landscape suitability (%) to Rift Valley fever outbreaks. The risk surface is derived from the ecological niche of RVF outbreaks using the Maxent model.

The predicted landscape suitability of the African continent and Arabian Peninsula to RVF outbreaks is presented in Fig 5. High risk landscapes were identified in Mauritania extending eastward into the Sahel, as well as in large portions of Sudan, Kenya, Tanzania, South Africa, Madagascar, and a corridor adjacent to the Red Sea in Saudi Arabia and Yemen. Moderate landscape suitability was predicted for northern parts of the Maghreb, the Horn of Africa and the broader Arabian Peninsula.

**Fig 6:**
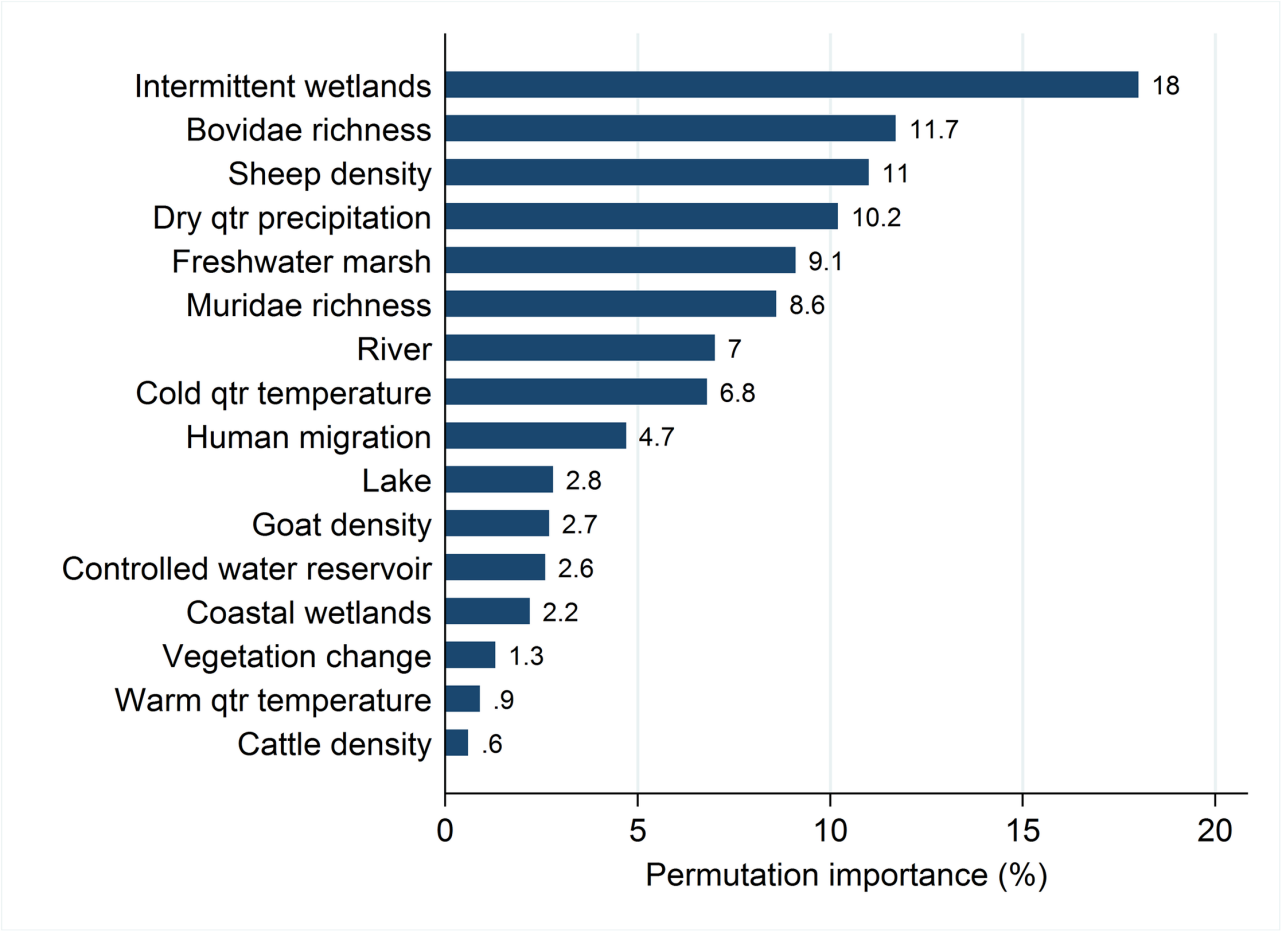
Permutation importance describing the relative importance of each feature to RVF landscape suitability as derived from the Maxent model.

The Maxent model identified proximity to intermittent wetlands (permutation importance = 18%), Bovidae species richness (11.7%), sheep density (11%), dry quarter precipitation (10.2%), and proximity to freshwater marsh (9.1%) as the most influential features to RVF landscape suitability (Fig 6). Muridae species richness was not as influential to suitability as Bovidae richness, but was impactful in the model with 8.6% permutation importance.

Response curves for these features and RVF outbreak risk are presented in S1 Fig. Increasing wild Bovidae species richness was associated with an increase in landscape suitability, as was sheep density up to an approximate average of 100 animals per km^2^, after which it decreased and remained constant at 250 animals per km^2^. Muridae richness demonstrated a V-shaped relationship with high landscape suitability in areas of low and high species richness. Close proximity to intermittent wetlands and freshwater marsh was associated with greater suitability to RVF outbreaks, as was low precipitation during the driest quarter. The model performed well when tested against the OIE data with the AUC equal to 83%.

## Discussion

This is the first study to explore and identify the influence of diverse surface water types on RVF outbreaks at a continental scale. The distribution of hydrogeographic features, particularly intermittent wetlands, contributed to suitable landscape conditions for RVF outbreaks. Our model also indicated that Bovidae species richness, sheep density and (to a lesser extent) Muridae species richness were predictive of RVF landscape suitability. This supports field observations that suggest that wild ruminants and rodents are the most likely wild reservoirs for RVFV [14]. These two important landscape features, i.e. wetland hydrogeography and Bovidae species richness, are novel contributions to our understanding of the epidemiology and infection ecology of RVF outbreaks in the African continent and the Arabian Peninsula.

Proximity to intermittent wetlands was particularly important to RVF landscape suitability, as identified by its permutation importance (18%). Hydrogeography has not previously been investigated across the diverse spectrum of surface water types in association with RVF occurrence in continent or country-wide analyses. One comprehensive study did find that proximity to rivers was an important determinant of landscape suitability across the African continent [43]. Our model, which is based on a larger number of epidemic occurrences, identified other wetland features–namely intermittent wetlands and freshwater marsh–as more important contributors to RVF risk. Nonetheless, the two continent-wide studies are in general agreement with respect to high risk landscapes in the Sahel, and eastern and southern Africa.

A regional study in the Ferlo area of Senegal examined proximity to transient ponds in hyperlocal settings of mixed vegetation cover and found that the juxtaposition of these small bodies of water with dense vegetation was strongly associated with positive serology in sheep and goats [44]. Moreover, this same group demonstrated that these landscape features corresponded to fluctuations in *Aedes* and *Culex* species during RVF epidemic years [45]. These kinds of transient surface water features were the most influential features to RVF landscape suitability in our model as well. Another study in the Mbeya region of Tanzania, found that proximity to Lake Malawi was very strongly associated with antibody evidence of past infection in humans [46,47], which is consistent with our finding of the importance of fixed freshwater sources such as freshwater marshes.

Our understanding of the landscape epidemiology of RVFV is, perhaps, most deficient with respect to reservoir hosts. Several potential mammalian RVFV hosts have been studied in both field and experimental settings to identify natural reservoirs in RVFV infection ecology [14]. While several species across multiple orders may be implicated as reservoirs, one review identified ruminants and rodents as likely natural reservoir(s) [14]. Evidence from the same study suggests that ruminants are more effective in virus amplification rather than maintenance and this may pose the greatest risk to humans in proximity. Consistent with this, our model demonstrated that wild Bovidae species richness was important in delineating the landscape suitability of RVF (permutation importance 11.7%). Interestingly, this landscape feature was as important as sheep density and more important than density of goats and cattle (see below). Muridae species richness was not as influential as Bovidae species richness, but the former did contribute to RVF landscape suitability (permutation importance = 8.6%). The V-shaped response curve also suggested high risk landscapes associated with low and high Muridae species richness and lower risk landscapes across intermediate species richness. While some previous work has identified the possibility of individual Muridae species as possible maintenance [14] or amplification hosts [48–54], the findings from the current study cannot attribute either role to the Muridae.

The density of sheep was strongly associated with landscape suitability to RVF outbreaks (permutation importance = 11%). This is not surprising given the high susceptibility of sheep [5] and their focus within most of the RVF outbreaks that occurred between 1998 and 2016 [55,56]. Moreover, it is through contact with animals and animal products that most human RVF occurs [13,57,58]. Goat and cattle density did not contribute substantively to RVF landscape suitability(permutation importance < 3 for both). While goats certainly have been affected in many RVF outbreaks, their lesser susceptibility relative to both sheep and cattle has been previously demonstrated [56].

Dry quarter precipitation was a moderately strong contributor to landscape suitability. Low levels of precipitation were associated with increased landscape suitability during the driest period of the year. However, as precipitation increased during this time, RVF suitability sharply decreased (S1 Fig). This may suggest that climatically drier areas are more susceptible when periodically inundated with sporadic episodes of rain [56]. Indeed, barren, arid landscapes punctuated with temporary ponds were associated with the greatest proliferation of both maintenance and amplification vector mosquitoes in West Africa [55,59]. Furthermore, regions with sensitive hydrogeographic dependency demonstrate high RVF susceptibility to precipitation variability attributable to El Nino southern oscillations [18,60]. Indeed, the latter, more recent, study identified a clear sensitivity to both seasonal climatic variation and El Nino oscillation across the whole of the African continent [60]. While the current findings may suggest climatic variations in arid to semi-arid conditions, or extremes of precipitation between dry and wet seasons, we emphasize that our study did not assess the relationship between RVF outbreaks and specific weather events.

This investigation identified landscapes suitable to RVFV beyond the historical extent of transmission. This suggests that conditions may be favorable for transmission should the virus be introduced into these naïve geographic spaces. In essence this allows us to conceive of how a realized niche in one space may be related to a potential niche in another, and what structures may be necessary or useful to prevent the realization of the niche in the novel space. For example, the introduction of RVFV from infected cattle herds to non-infected susceptible herds has been well documented in both local [61] and global [62,63] scales. More specifically, network structure and resulting dissemination of herd animals across villages has been shown to drive the introduction or re-introduction of RVFV to susceptible livestock herds [61]. Translocations of RVFV at more global scales can follow international trade in livestock [62], as well national-level trade in livestock [63]. Surveillance mechanisms should target transboundary livestock movement and mosquito control along livestock migratory corridors for effective prevention of RVF transmission. Indeed, some success has been documented with respect to cross-disciplinary, systems-based approaches to RVFV surveillance in East Africa as organized by the Armed Forces Health Surveillance Center, Division of Global Emerging Infections Surveillance and Response System Operations (AFHSC-GEIS)[64].

A perennial problem in ecological niche modeling, in general, and in emerging disease risk mapping, in particular, is the lack of independent sample data for testing trained models [65]. Typically only one sample is available, which is partitioned into training and testing datasets. A strength of the current study is that we utilized two distinct sources of RVF outbreak data; one (ProMED) to train the landscape suitability model and the other (OIE) to test the prediction error of the model. Nonetheless, the findings from the current study must be interpreted with caution due to its limitations. First, temperature and precipitation were measured as single composites over the period 1950 to 2000. Therefore, while the spatial resolution of these measurements was high (~1 km^2^ rasters), the analysis was simultaneously constrained by coarse temporal resolution, given that the climate rasters represent 50 year averages. Second, the number of documented RVF occurrences is small. Therefore, with only 103 ProMED-reported occurrences and 78 unique OIE-reported occurrences, this collection of RVF outbreaks may not represent the true incidence across the continent of Africa and the Arabian Peninsula. Furthermore, the model may produce an underfit representation of the ecological niche of RVF. Nevertheless, the current analysis attempted a robust validation of models trained on limited data by using a second vetted source of RVF occurrence data from OIE for testing. Third, due to a lack of adequate presence data, the current investigation was unable to evaluate the influence of *Aedes* and *Culex* mosquitoes. Because these vectors are part of the RVFV infection cycle, the lack of *Aedes* and *Culex* mosquitoes in our model necessarily demarcates a less refined picture of RVF landscape suitability. Nevertheless, the hydrogeographic features identified by the model as influential to RVF suitability correspond to surface water features documented to be most favorable to the *Aedes* mosquitoes [44,45,66] and *Culex* mosquitoes [2,59,67] and so the model still appears to identify landscapes appropriate to the relevant vector ecology.

The findings of this study will help to improve our understanding of the landscape epidemiology of RVF outbreaks. The model of RVF landscape suitability will require further development as more data become available to validate the results. Moreover, because this study is observational rather than experimental, we cannot assign causation to the associations between RVF outbreaks and landscape factors, including surface water features and mammalian hosts. More definitive causal inference demands better assessment of species in the landscapes where outbreaks are occurring, and crucially at high spatial resolution and in real time. This will require thorough field studies that combine animal serology and viral RNA detection, more detailed habitat description, observation of wild Bovidae movement patterns, wildlife-domestic animal interaction, and maintenance and amplification vectors, as well as cultural and economic drivers of the livestock industries.

### Conclusion

This study found that proximity to wetlands in landscapes that are rich in wild Bovidae species and high in sheep density, delineate the most suitable landscapes for RVF outbreaks. Moreover, this study found that the RVF risk surface may extend to regions beyond the historical range of past zoonotic experience should the virus be introduced to these regions via livestock transport or local invasion by infected mosquitoes.

## Supporting information

S1 Fig Variable response curves for the most influential landscape factors in identifying the landscape suitability of Rift Valley fever outbreaks based on the Maxent model. (PNG)
