# Supplementary figures and images for: Wetlands, wild Bovidae species richness and sheep density delineate risk of Rift Valley fever outbreaks in the African continent and Arabian Peninsula
Source: PLoS Negl Trop Dis. 2017 Jul 25;11(7):e0005756. doi: 10.1371/journal.pntd.0005756 (PMC5526521; doi:10.1371/journal.pntd.0005756)

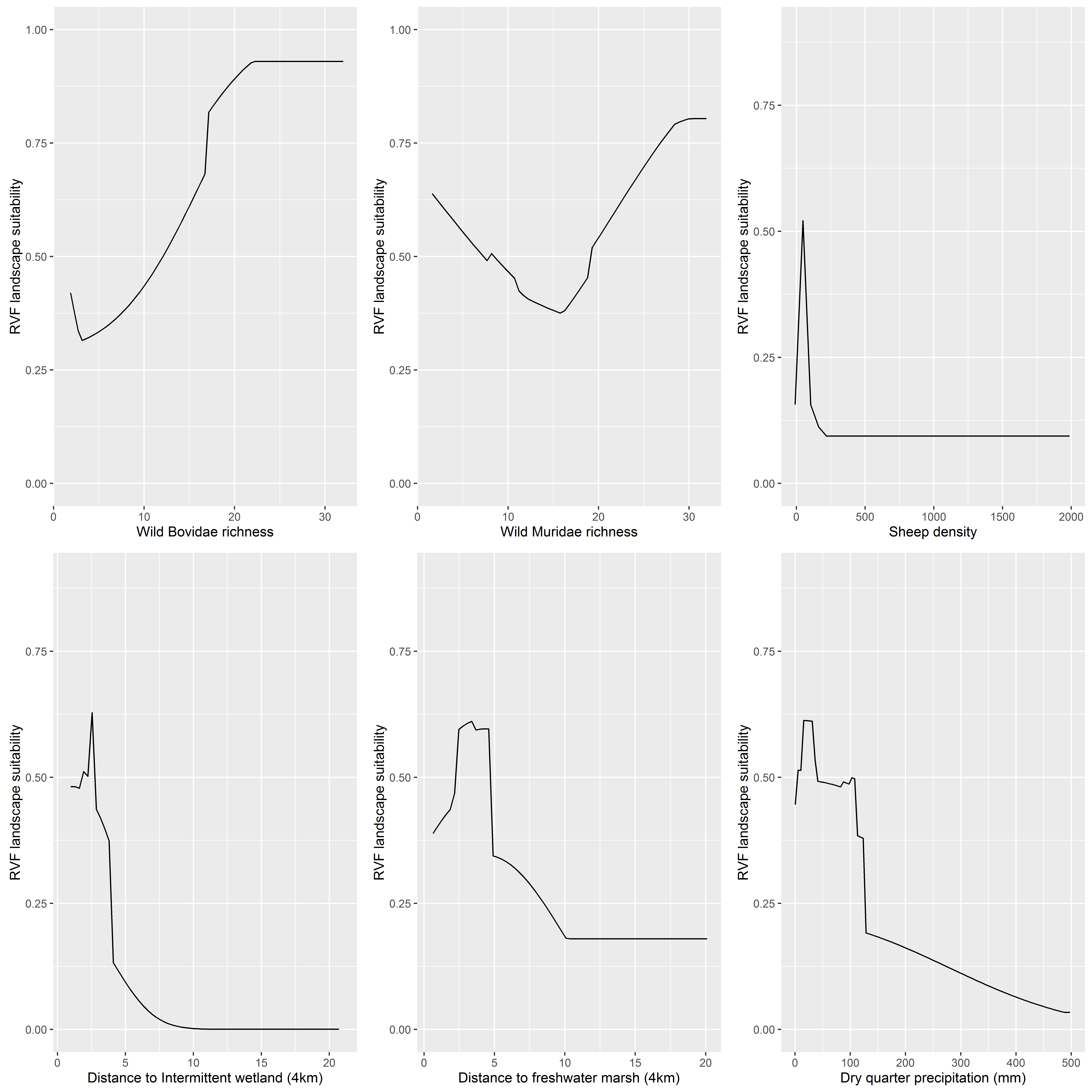

Supplement: S1 Fig — (PNG) [file pntd.0005756.s001.png]
